# Trilaciclib prior to chemotherapy and atezolizumab in patients with newly diagnosed extensive‐stage small cell lung cancer: A multicentre, randomised, double‐blind, placebo‐controlled Phase II trial

**DOI:** 10.1002/ijc.33453

**Published:** 2021-01-12

**Authors:** Davey Daniel, Vladimer Kuchava, Igor Bondarenko, Oleksandr Ivashchuk, Sreekanth Reddy, Jana Jaal, Iveta Kudaba, Lowell Hart, Amiran Matitashvili, Yili Pritchett, Shannon R. Morris, Jessica A. Sorrentino, Joyce M. Antal, Jerome Goldschmidt

**Affiliations:** ^1^ Sarah Cannon Research Institute, Tennessee Oncology‐Chattanooga Chattanooga Tennessee USA; ^2^ LTD Institute of Clinical Oncology Tbilisi Georgia; ^3^ Dnipropetrovsk Medical Academy Dnipro Ukraine; ^4^ Chernivtsi Regional Clinical Oncology Centre Chernivtsi Ukraine; ^5^ Northside Hospital, Inc Atlanta Georgia USA; ^6^ Department of Hematology‐Oncology University of Tartu Tartu Estonia; ^7^ Latvian Oncology Centre Riga East University Hospital Riga Latvia; ^8^ Florida Cancer Specialists Fort Myers Florida USA; ^9^ LTD Cancer Research Centre Tbilisi Georgia; ^10^ G1 Therapeutics Research Triangle Park North Carolina USA; ^11^ Blue Ridge Cancer Care Blacksburg Virginia USA

**Keywords:** chemotherapy, myelopreservation, myelosuppression, small cell lung cancer (SCLC), trilaciclib

## Abstract

Trilaciclib is an intravenous CDK4/6 inhibitor administered prior to chemotherapy to preserve haematopoietic stem and progenitor cells and immune system function from chemotherapy‐induced damage (myelopreservation). The effects of administering trilaciclib prior to carboplatin, etoposide and atezolizumab (E/P/A) were evaluated in a randomised, double‐blind, placebo‐controlled Phase II study in patients with newly diagnosed extensive‐stage small cell lung cancer (ES‐SCLC) (NCT03041311). The primary endpoints were duration of severe neutropenia (SN; defined as absolute neutrophil count <0.5 × 10^9^ cells per L) in Cycle 1 and occurrence of SN during the treatment period. Other endpoints were prespecified to assess the effects of trilaciclib on additional measures of myelopreservation, patient‐reported outcomes, antitumour efficacy and safety. Fifty‐two patients received trilaciclib prior to E/P/A and 53 patients received placebo. Compared to placebo, administration of trilaciclib resulted in statistically significant decreases in the mean duration of SN in Cycle 1 (0 vs 4 days; *P* < .0001) and occurrence of SN (1.9% vs 49.1%; *P* < .0001), with additional improvements in red blood cell and platelet measures and health‐related quality of life (HRQoL). Trilaciclib was well tolerated, with fewer grade ≥3 adverse events compared with placebo, primarily due to less high‐grade haematological toxicity. Antitumour efficacy outcomes were comparable. Administration of trilaciclib vs placebo generated more newly expanded peripheral T‐cell clones (*P* = .019), with significantly greater expansion among patients with an antitumour response to E/P/A (*P* = .002). Compared with placebo, trilaciclib administered prior to E/P/A improved patients' experience of receiving treatment for ES‐SCLC, as shown by reduced myelosuppression, and improved HRQoL and safety profiles.

**Abbreviations**
AE: adverse events
ASCO: American Society of Clinical Oncology
CI: confidence intervals
CIM: chemotherapy‐induced myelosuppression
CTCAE: Common Terminology Criteria for Adverse Events
DBL: database locks
DOR: duration of objective response
DSN: duration of severe neutropenia
ECOG PS: Eastern Cooperative Oncology Group performance status
ESA: erythropoiesis‐stimulating agents
FACT: Functional Assessment of Cancer Therapy
FDA: Food and Drug Administration
FN: febrile neutropenia
FWB: functional well‐being
HR: hazard ratio
HRQoL: health‐related quality of life
HSPC: haematopoietic stem and progenitor cells
ICI: immune checkpoint inhibitors
ITT: intention‐to‐treat
ORR: objective response rate
OS: overall survival
PBMC: peripheral blood mononuclear cells
PFS: progression‐free survival
PRO: patient‐reported outcomes
PWB: physical well‐being
RBC: red blood cell
RDI: relative dose intensity
SAE: serious adverse events
SCLC: small cell lung cancer
SN: severe neutropenia
TCR: T‐cell receptor
TNBC: triple‐negative breast cancer
TTD: time to deterioration
WBC: white blood cell

**What's new**

- Trilaciclib is known to protect immune function and blood progenitor cells from chemotherapy‐induced damage. Might it improve outcomes for patients with small‐cell lung cancer (SCLC)? In this prospective, randomized study, the authors found that when trilaciclib was given prior to treatment with chemotherapy plus atezolizumab, it reduced myelosuppression and the need for supportive care. It also enhanced T‐cell immunity, and improved quality of life. Trilaciclib thus has considerable potential as a new standard of supportive care for SCLC patients receiving myelosuppressive chemotherapy.

## 1 INTRODUCTION

The use of platinum chemotherapy (cisplatin or carboplatin) in combination with etoposide for the treatment of extensive‐stage small cell lung cancer (ES‐SCLC)^1^ can damage haematopoietic stem and progenitor cells (HSPCs) in the bone marrow, resulting in significant chemotherapy‐induced myelosuppression (CIM) that manifests as neutropenia, anaemia and thrombocytopenia.^2^, ^3^, ^4^, ^5^, ^6^, ^7^ The adverse consequences of chemotherapy are particularly relevant in SCLC, where more than half of patients are aged ≥65 years at diagnosis and patients often present with multiple comorbidities. As such, patients with SCLC are more likely to experience clinically significant side effects related to CIM.^8^, ^9^ The haematological toxicities of CIM are a major source of morbidity, mortality and cost among patients with cancer, due to an increased risk of infection, sepsis, bleeding and fatigue. Currently, CIM is managed with dose delays and reductions that reduce the dose intensity and potentially, the antitumour efficacy, of chemotherapy, as well as intervention with growth factors or transfusions.^10^ Consequently, preventing CIM is an important goal in the treatment of patients with cancer.

Chemotherapy remains a major component of treatment for patients with SCLC; however, despite high initial response rates, long‐term outcomes remain poor. Cytotoxic drugs can cause tumour cells to become more susceptible to immune destruction by stimulating antigen presentation and T‐cell priming.^11^ However, tumour cells have developed multiple mechanisms for evading immune surveillance. Combining immune checkpoint inhibitors (ICIs) with chemotherapy may disrupt these escape mechanisms and efficiently restore the antitumour activity of the immune system.^12^ Considering the success of ICI for the treatment of non‐SCLC,^12^ research into whether ICI could also provide durable responses and improved survival in patients with SCLC became a matter of priority. The anti–programmed death ligand‐1 (PD‐L1) antibody, atezolizumab, in combination with etoposide and carboplatin (E/P) was approved by the U.S. Food and Drug Administration (FDA) in 2019 for the first‐line treatment of patients with ES‐SCLC. Approval was based on results from the IMpower133 trial, which showed that atezolizumab plus chemotherapy significantly improved overall survival (OS) vs chemotherapy alone.^13^ More recently, the PD‐L1 antibody, durvalumab, was also approved by the FDA in combination with etoposide plus platinum chemotherapy as a first‐line treatment for adult patients with ES‐SCLC, based on the results of the Phase III CASPIAN trial.^14^ Although the combination of chemotherapy plus ICIs may provide improved survival for some patients, further research is required to optimise treatment outcomes for patients with ES‐SCLC.

Chemotherapy indiscriminately kills proliferating cells, including HSPCs and immune cells, and therefore the full benefit of chemotherapy plus ICI combinations may not be realised due to resulting CIM and immunosuppression.^15^ An intervention that both prevents CIM and maintains immune system function when cytotoxic treatment is administered could therefore reduce the adverse consequences of chemotherapy and potentially augment the antitumour efficacy of chemotherapy plus ICI combination regimens.

Trilaciclib is being developed as a first‐in‐class myelopreservation therapy to prevent CIM in adult patients with ES‐SCLC.^16^, ^17^ Trilaciclib is an intravenous (IV) CDK4/6 inhibitor that transiently arrests CDK4/6‐dependent cells, including HSPCs and lymphocytes, in the G1 phase of the cell cycle, thereby preventing them from proliferating in the presence of cytotoxic chemotherapy. In doing so, trilaciclib provides resistance to chemotherapy‐induced damage, and favourably alters the tumour immune microenvironment through transient T‐cell inhibition, with differential effects on T‐cell subsets.^16^, ^17^, ^18^ The myelopreservation benefits of trilaciclib have been evaluated in patients receiving chemotherapy for the treatment of SCLC because SCLC tumour cells replicate independently of CDK4/6 due to the obligate loss of retinoblastoma. This allows the assessment of trilaciclib's effects on the host while minimising theoretical concerns related to effects on the tumour.^19^

In addition to protecting lymphocyte populations and increasing immune activation through differential T‐cell recovery, trilaciclib and other CDK4/6 inhibitors have been shown to enhance antitumour responses through other mechanisms in preclinical models, including enhanced T‐cell activation through modulation of nuclear factor of activated T‐cell activity and upregulation and stabilisation of PD‐L1 expression on tumour cells, resulting in increased sensitivity to ICI.^18^, ^20^ Preclinically, the addition of trilaciclib to chemotherapy/ICI combinations has been shown to enhance and prolong the duration of the antitumour responses, providing a rationale for combining trilaciclib with chemotherapy/ICI regimens in patients with cancer.^18^

Data from a randomised, double‐blind, placebo‐controlled Phase II trial of trilaciclib administered prior to E/P therapy in patients with newly diagnosed ES‐SCLC showed myelopreservation benefits across multiple haematopoietic lineages (neutrophils and red blood cells [RBCs]) compared with placebo, with patients requiring fewer supportive care interventions and chemotherapy dose reductions.^21^ The current study was designed to confirm the effects of trilaciclib on CIM in patients with newly diagnosed ES‐SCLC treated with E/P and to investigate if the immune‐enhancing effects of trilaciclib would translate to an improvement in the antitumour efficacy of atezolizumab.

## 2 PATIENTS AND METHODS

### 2.1 Study design and participants

This was a global, randomised, double‐blind, placebo‐controlled, multicentre Phase II study (NCT03041311; EudraCT 2017‐000358‐20). Eligible patients were ≥18 years of age with confirmed ES‐SCLC, an Eastern Cooperative Oncology Group performance status (ECOG PS) of 0‐2 and measurable disease by Response Evaluation Criteria in Solid Tumours Version 1.1 (RECIST v1.1). Patients were ineligible for inclusion if they presented with symptomatic brain metastases or had received prior systemic therapy for limited‐stage or ES‐SCLC (see Supplementary Methods for full inclusion/exclusion criteria).

### 2.2 Randomisation and procedures

Patients were randomised 1:1 in a blinded manner to receive trilaciclib or placebo prior to etoposide, carboplatin and atezolizumab (E/P/A) therapy. An interactive web‐response system was used to randomise patients according to a randomisation schedule generated by an unblinded statistician. Randomisation was stratified by ECOG PS (0/1 vs 2) and the presence of brain metastases (yes or no).

Patients were treated in an induction phase and a maintenance phase. During induction, patients received trilaciclib or placebo prior to E/P/A for a maximum of four 21‐day cycles. Trilaciclib (240 mg/m^2^) or placebo was administered as a 30‐minute IV infusion, once daily on day (D) 1, D2 and D3, prior to E/P/A therapy. Etoposide (100 mg/m^2^) was administered on D1, D2 and D3, and carboplatin (at area under the concentration‐time curve 5; maximum dose 750 mg) on D1, both via IV infusion. Atezolizumab (1200 mg) was administered on D1 by IV infusion, following completion of E/P administration. During maintenance, patients received atezolizumab monotherapy on D1 of every 21‐day cycle; neither trilaciclib nor placebo was administered. No dose modifications of trilaciclib or atezolizumab were allowed. Per protocol, dose reductions of E/P (both reduced at same time) were allowed twice for any patient and were permanent. Treatment could continue until disease progression per RECIST v1.1, unacceptable toxicity, withdrawal of consent, or discontinuation by investigator, whichever occurred first. Following radiographic disease progression per RECIST v1.1, if the patient appeared to be deriving clinical benefit, the investigator believed it was in the best interest of the patient and the patient had provided reconsent, study drug administration could be continued until loss of clinical benefit. Standard‐of‐care supportive interventions, including RBC and platelet transfusions, were allowed per investigator discretion throughout the entire treatment period. Primary prophylaxis with granulocyte colony‐stimulating factors (G‐CSFs) and use of erythropoiesis‐stimulating agents (ESAs) were prohibited in cycle (C) 1 of induction, although therapeutic G‐CSF was allowed in all cycles. Per standard guidelines, the risk of FN with E/P is ≤20% and primary prophylaxis with G‐CSF is not recommended unless the investigator determines the patient has individual clinical characteristics that increase the FN risk to >20%. Therefore, per the guidelines, investigators determined whether to enrol patients with the understanding that primary prophylaxis with G‐CSF was prohibited in C1. Following completion of C1, both ESAs and G‐CSF (prophylactic and therapeutic) were allowed per American Society of Clinical Oncology (ASCO) standard‐of‐care guidelines.^22^

### 2.3 Outcomes

The primary objective of the study was to evaluate the myelopreservation efficacy of trilaciclib vs placebo when administered prior to myelosuppressive chemotherapy plus atezolizumab. All myelopreservation analyses included laboratory value data rather than adverse events (AEs), unless otherwise specified. Primary endpoints were the duration of severe neutropenia (DSN) in C1 and percentage of patients with severe neutropenia (SN) (occurrence) during the treatment period. SN was defined as absolute neutrophil count (ANC) <0.5 × 10^9^ cells per L.

Key secondary endpoints included occurrence of RBC transfusions on or after Week 5, occurrence of G‐CSF administrations and total number of all‐cause chemotherapy dose reductions. Supportive secondary myelopreservation endpoints included occurrence of Grade 3 or 4 haematological laboratory abnormalities, febrile neutropenia (FN) AEs, ESA administrations, platelet transfusions, IV antibiotic administration and infection serious adverse events (SAEs). Supportive antitumour efficacy endpoints included objective response rate (ORR), duration of objective response (DOR), progression‐free survival (PFS) and OS.

Assessment of exploratory endpoints measuring trilaciclib's effects on health‐related quality of life (HRQoL) was based on validated Functional Assessment of Cancer Therapy (FACT) instruments (FACT‐General, FACT‐Lung and FACT‐Anemia), using literature‐based thresholds of meaningful within‐patient change.^23^, ^24^, ^25^, ^26^ Confirmed deterioration in patient‐reported outcomes (PROs) was defined as a decrease from baseline by a clinically meaningful threshold for two consecutive visits; that is, change from baseline of ≤−3 points for physical well‐being (PWB), social well‐being (SWB), emotional well‐being (EWB), functional well‐being (FWB), lung cancer subscale (LCS) and fatigue; ≤−6 points for FACT‐Lung, lung trial outcome index and anaemia trial outcome index points, and ≤−7 points for FACT‐General and FACT‐Anemia total scores.

Safety was evaluated by measuring the occurrence and severity of AEs by National Cancer Institute Common Terminology Criteria for Adverse Events, Version 4.03. The occurrence and incidence (per 100 cycles) of hospitalisation (all cause and due to CIM [neutropenia, anaemia, thrombocytopenia] or sepsis), chemotherapy exposure, dose reductions, and dose interruptions (defined as infusion interruptions) were evaluated as part of the safety assessments.

### 2.4 PD‐L1 immunohistochemistry

For the PD‐L1 analysis, archival tissue was collected from each patient. The VENTANA PD‐L1 (SP142) immunohistochemical assay (Roche) was used to assess expression of PD‐L1, using the rabbit monoclonal anti‐PD‐L1 clone SP142. An IgG antibody was used as a negative control and human tonsil tissue as a positive control. Digital images of each section were generated using an Aperio Scanscope. Samples were considered negative or positive if <1% or ≥1% of the total tumour area (including stroma and inflammatory regions) contained PD‐L1‐labelled immune cells, respectively. All analyses were completed by Epistem, Ltd (Manchester, UK).

### 2.5 Flow cytometry analysis

The ability of trilaciclib to preserve immune system function was assessed by measuring change from baseline in immune cell subsets. Specifically, levels of activated CD8+ T cells, activated Th1 cells and regulatory T cells (Tregs) were analysed via flow cytometric analysis of blood at baseline (iC1D1), start of maintenance (mC1D1), during maintenance (mC5D1) and 90 days after the end of treatment (PTV +90) (Supplementary Methods). Flow cytometric analysis was completed at Covance Central Laboratory Services, Inc (Geneva, Switzerland and Indianapolis, US), with statistical analyses performed by Fios Genomics Ltd (Edinburgh, UK).

### 2.6 T‐cell receptor β CDR3 analysis

To assess the effect of trilaciclib on the peripheral T‐cell compartment and clonal expansion, T‐cell receptor (TCR) β CDR3 regions were amplified and sequenced from purified genomic DNA in peripheral blood mononuclear cells isolated from whole blood samples at iC1D1 and mC1D1 using the immunoSEQ Assay (Adaptive Biotechnologies, Seattle, US) (Supplementary Methods). Newly detected expanded clones were defined as clones that were not detected at baseline, but were measurable at mC1D1.

### 2.7 Statistical analysis

The planned sample size of the study was 106 (approximately 53 per group). Sample size was calculated to support the evaluation of trilaciclib prior to E/P/A vs placebo prior to E/P/A on each of the primary endpoints, with at least 90% power at a two‐sided significance level of .025 (Bonferroni split of overall 2‐sided α = .05 between the two primary endpoints). The assumed treatment effects on DSN in C1 and occurrence of SN were a between‐group mean difference of 2 days (SD 2.5), and an absolute reduction of 34% (assuming a placebo event rate of 45%), respectively. The sample size was adjusted for the possibility that 5% of patients would not have any postbaseline ANC assessments.

The intention‐to‐treat (ITT) analysis set, used for myelopreservation, PROs and PFS/OS endpoints, included all randomised patients, with data analysed by randomly assigned treatment. Safety analyses included all randomised patients who received at least one dose of study drug, with data analysed by actual received treatment. Analyses of tumour response were performed in patients who had a measurable target lesion at baseline, and had either at least one postbaseline tumour assessment, discontinued treatment due to clinical progression or died due to disease progression before their first postbaseline tumour scan. There were two clinical database locks (DBLs); myelosuppression and PRO endpoints were analysed based on the data from the first DBL, which took place when all randomised patients had finished at least 12 weeks of treatment or discontinued study drug prior to Week 12 (data cutoff: August 17, 2018). Analyses for myelosuppression endpoints and PRO endpoints with respect to continuous and categorical change from baseline included data from the induction phase only, while the analysis of time to deterioration for PRO endpoints included all data (induction and maintenance) available at the time of the data cutoff for the first DBL. Data from the second DBL (data cutoff: June 28, 2019) were used for safety and antitumour efficacy evaluation.

DSN in C1 was evaluated using a nonparametric analysis of covariance.^27^ Patients who did not have SN in C1 were assigned a value of 0. For binary endpoints (eg, occurrence of SN or RBC transfusion on or after Week 5), treatment effect was assessed using a modified Poisson regression model.^28^ For counting endpoints, a negative binomial regression model was used to evaluate treatment effect. All statistical models included treatment, ECOG PS (0/1 vs 2) and brain metastases (yes or no) as fixed effects, with corresponding baseline value as a covariate. For the primary and key secondary efficacy endpoints, a Hochberg‐based gatekeeping procedure^29^ was utilised to allow strong control of family‐wise Type I error rate at one‐sided α = .025 level. Mean difference in DSN in C1, with one‐sided, multiplicity‐adjusted *P* values and 95% confidence intervals (CI) is reported. Adjusted relative risk (aRR) and 95% CI are reported for all other binary and counting endpoints.

A post hoc analysis of DSN in C1, occurrence of SN and occurrence of RBC transfusion on or after Week 5 was evaluated by age subgroup (<65 and ≥65 years). The same statistical models were applied to each group to estimate the treatment effect of trilaciclib vs placebo.

Tumour response status per RECIST v1.1 was derived from measurements provided by the investigator. ORR and its exact 95% CI using the Clopper‐Pearson method were computed for each treatment group. The treatment effect was evaluated using a stratified Cochran‐Mantel‐Haenszel method. DOR was characterised using the Kaplan‐Meier method for patients who achieved a complete or partial response. The Kaplan‐Meier method was used to estimate median PFS and OS; treatment group difference was evaluated using a stratified log‐rank test, with the hazard ratio (HR) and its 95% CI generated from a Cox proportional hazard model. OS data are considered mature when at least 70% of deaths have occurred (not reached at the time of the second DBL). Safety measures are summarised using descriptive statistics, except for hospitalisation due to CIM or sepsis, where treatment group differences were assessed using a modified Poisson model and incidence rates using a negative binomial model. All statistical analyses were conducted using SAS software, v.9.4.

## 3 RESULTS

### 3.1 Patient disposition, demographics and baseline disease characteristics

Between June 29, 2017 and February 9, 2018, 125 patients were enrolled in the study. Of these, 107 were eligible and randomly assigned to the trilaciclib group (n = 54) or the placebo group (n = 53; ITT population; Supplementary Figure S1). Two patients were randomised to receive trilaciclib but did not receive any study drug (one patient did not meet eligibility criteria and was randomised in error and one patient's platelet count did not meet dosing criteria on C1D1). Baseline demographics and disease characteristics were similar between the treatment groups. Expression of PD‐L1 was detected in 18/48 (37.5%) tumour tissue samples, including 8/21 (38.1%) in the trilaciclib group and 10/27 (37.0%) in the placebo group (Table 1).

**TABLE 1:** Patient demographic and baseline disease characteristics

Measure		Trilaciclib prior to E/P/A	Placebo prior to E/P/A
		(n = 54)	(n = 53)
Age, years	Median (range)	65 (45‐81)	64 (46‐83)
	18 to <65, n (%)	27 (50.0)	27 (50.9)
	≥65, n (%)	27 (50)	26 (49)
Sex, n (%)	Male	41 (75.9)	34 (64.2)
	Female	13 (24.1)	19 (35.8)
Race, n (%)	White	53 (98.1)	51 (96.2)
	Black or African American	0	1 (1.9)
	Other	1 (1.9)	1 (1.9)
Region, n (%)	USA	20 (37.0)	22 (41.5)
	Non‐USA	34 (63.0)	31 (58.5)
ECOG PS, n (%)	0–1	45 (85.2)	46 (86.8)
	2	8 (14.8)	7 (13.2)
Baseline LDH, n (%)	≤ULN	26 (48.1)	29 (54.7)
	>ULN	25 (46.3)	24 (45.3)
	Missing	3 (5.6)	0
Brain metastases, n (%)		15 (27.8)	15 (28.3)
Smoking history, n (%)	Never smoked	4 (7.4)	6 (11.3)
	Former smokers	26 (48.1)	29 (54.7)
	Current smokers	23 (42.6)	18 (34.0)
	Missing	1 (1.9)	0
PD‐L1 status,^a^ n/n (%)	Negative	13/21 (61.9)	17/27 (63.0)
	Positive	8/21 (38.1)	10/27 (37.0)

*Notes:* Intention‐to‐treat population.

Abbreviations: ECOG PS, Eastern Cooperative Oncology Group performance status; E/P/A, etoposide, carboplatin and atezolizumab; LDH, lactate dehydrogenase; PD‐L1, programmed death ligand‐1; ULN, upper limit of normal.

^a^ Assessed using the VENTANA PD‐L1 (SP142) immunohistochemical assay; samples were considered negative or positive if <1% or ≥1% of the total tumour area (including stroma and inflammatory regions) contained PD‐L1–labelled immune cells, respectively.

### 3.2 Myelopreservation

**FIGURE 1:**
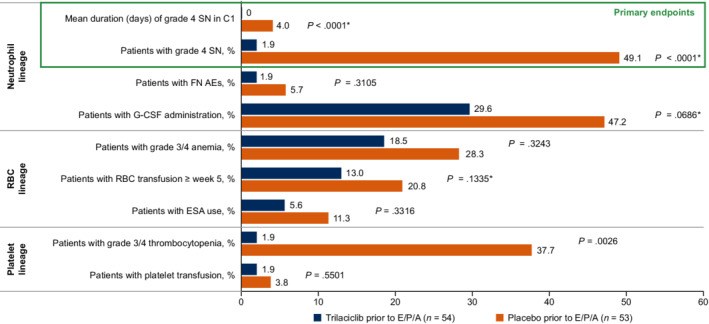
Summary of myelosuppression endpoints. Data are from the induction phase. *P* values are raw one‐sided or multiplicity‐adjusted. *Multiplicity adjusted *P* value. AEs, adverse events; C, cycle; E/P/A, etoposide, carboplatin and atezolizumab; ESA, erythropoiesis‐stimulating agent; FN, febrile neutropenia; G‐CSF, granulocyte colony‐stimulating factor; RBC, red blood cell; SN, severe neutropenia

Trilaciclib administered prior to E/P/A therapy reduced chemotherapy‐induced neutropenia compared with placebo, as measured by statistically significant improvements in the primary endpoints of DSN in C1 and occurrence of SN (Figure 1; Supplementary Table S1). Mean DSN was 0 days (SD, 1.0) with trilaciclib vs 4 days (4.7) with placebo (mean difference [95% CI] −3.6 days [−4.9, −2.3]; raw and multiplicity‐adjusted *P* value <.0001). One patient (1.9%) had SN with trilaciclib vs 26 patients (49.1%) with placebo (aRR [95% CI] 0.038 [0.008, 0.195], raw and multiplicity‐adjusted *P* value <.0001). Trilaciclib administered prior to E/P/A therapy also reduced the need for RBC transfusions (aRR [95% CI] 0.642 [0.294, 1.404]; raw and multiplicity‐adjusted *P* value = .1335) and use of G‐CSF (aRR [95% CI] 0.646 [0.403, 1.034]; raw *P* value = .0343, multiplicity‐adjusted *P* value = .0686) (Figure 1; Supplementary Table S1). The rate of all‐cause chemotherapy dose reductions (calculated as events per 100 cycles) was significantly lower in the trilaciclib group vs the placebo group (2.1 vs 8.5, respectively; raw *P* value = .0195, multiplicity‐adjusted *P* value = .0065). There were also reductions in the use of platelet transfusions and ESAs with trilaciclib compared with placebo, although differences between the treatment groups were not statistically significant. The evaluation of complete blood cell counts showed that compared with placebo, patients receiving trilaciclib had higher ANC nadirs, a slower decline in haemoglobin over time and similar mean platelet and lymphocyte counts (Supplementary Figure S2).

Four patients experienced FN AEs during induction, including 1 (1.9%) patient in the trilaciclib group and 3 (5.7%) patients in the placebo group. Infection SAEs occurred in 3 (5.6%) patients in the trilaciclib group and 7 (13.2%) patients in the placebo group. IV antibiotics were required in 10 (18.5%) patients in the trilaciclib group and 12 (22.6%) patients in the placebo group.

Subgroup analyses showed that trilaciclib consistently reduced mean DSN in C1 and occurrence of SN compared with placebo in patients aged <65 and ≥65 years (Supplementary Table S2). For patients aged ≥65 years, RBC transfusions on or after Week 5 occurred in 5 (18.5%) patients treated with trilaciclib and 10 (38.5%) patients treated with placebo. Two patients aged <65 years required an RBC transfusion on or after Week 5 with trilaciclib vs one patient with placebo.

### 3.3 Patient experience

**FIGURE 2:**
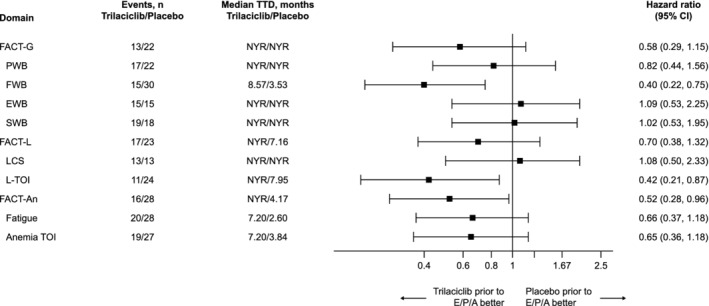
Median time to confirmed deterioration in patient‐reported outcomes. Data are from the time of DBL1. DBL1, first database lock (data cutoff: August 17, 2018); EWB, emotional well‐being; FACT‐An, Functional Assessment of Cancer Therapy‐Anemia; FACT‐G, Functional Assessment of Cancer Therapy‐General; FACT‐L, Functional Assessment of Cancer Therapy‐Lung; FWB, functional well‐being; LCS, Lung Cancer Subscale; NYR, not yet reached; PWB, physical well‐being; SWB, social well‐being; TOI, trial outcome index; TTD, time to deterioration

PRO completion rates were high (>92% in both groups) throughout the study. At the end of C4, there were no differences between the treatment groups in the adjusted mean difference from baseline for total or any subscale score. A trend favouring trilaciclib was observed at the end of C2 for several domains. For FWB, the least squares mean change from baseline (SE) was 2.02 (0.84) for trilaciclib and −0.17 (0.85) for placebo, with a HR for the difference in change from baseline between treatment groups (95% CI) of 2.19 (1.02). Responder analysis showed that patients receiving trilaciclib had higher improvement rates and lower deterioration rates than those receiving placebo, especially for FWB, PWB, anaemia and fatigue. With the exception of EWB, SWB and LCS domains, median time to deterioration for patients receiving trilaciclib was longer than for patients receiving placebo (HR range: 0.40‐0.82; Figure 2). Median time to deterioration in fatigue among patients receiving trilaciclib was 4.6 months longer than the median time to deterioration for patients receiving placebo (HR = 0.66 [95% CI: 0.37, 1.18]).

### 3.4 Safety

Most patients in both treatment groups completed all 4 cycles of induction therapy (44 [84.6%] patients in the trilaciclib group and 48 [90.6%] patients in the placebo group). Across both induction and maintenance, patients in both groups completed a median 8 treatment cycles (range: 2‐29 with trilaciclib and 1‐26 with placebo, respectively). During induction, median relative dose intensity (RDI) of E/P was 98.1% (E) and 98.8% (P) in the trilaciclib group and 92.3% (E) and 93.3% (P) in the placebo group, respectively. Median RDI of atezolizumab during induction and maintenance was 96.3% in the trilaciclib group and 95.0% in the placebo group. E/P dose reductions during induction occurred in 3 (5.8%) patients and 1 (1.9%) patient in the trilaciclib group and in 14 (26.4%) patients and 13 (24.5%) patients in the placebo group, respectively. The primary reason for chemotherapy dose reductions was haematological toxicity. Chemotherapy cycle delays occurred in 18 (34.6%) patients in the trilaciclib group and 31 (58.5%) patients in the placebo group.

There were fewer Grade 3 or 4 AEs with trilaciclib compared with placebo (Table 2), largely due to fewer Grade 3 or 4 haematological AEs in the trilaciclib group (19 [36.5%]) vs the placebo group (39 [73.6%]). Among patients receiving trilaciclib prior to E/P/A, 2 (3.8%) patients were hospitalised for CIM or sepsis, compared with 6 (11.3%) patients receiving placebo (*P* = .1287) (Supplementary Table S3). The incidence of hospitalisation due to CIM or sepsis was 1.03/100 cycles with trilaciclib vs 5.50/100 with placebo (Supplementary Table S3). Fifteen patients had AEs considered related to trilaciclib. The most common (≥5% of patients) AEs considered related to trilaciclib were fatigue (5 [9.6%] patients), nausea (4 [7.7%] patients), anaemia (3 [5.8%] patients) and infusion‐related reaction (3 [5.8%] patients). Trilaciclib‐related AEs were all Grade 1 or 2, with the exception of 3 (5.8%) patients who had Grade 3 events.

**TABLE 2:** Summary of adverse events occurring in ≥10% of all patients

		Trilaciclib prior to E/P/A			Placebo prior to E/P/A		
		(n = 52)			(n = 53)		
		All grades	Grade 3	Grade 4	All grades	Grade 3	Grade 4
Any AE, n (%)		49 (94.2)	23 (44.2)	6 (11.5)	52 (98.1)	15 (28.3)	26 (49.1)
Haematological, n (%)^a^	Anaemia	19 (36.5)	9 (17.3)	0	33 (62.3)	15 (28.3)	1 (1.9)
	Neutropenia	19 (36.5)	9 (17.3)	1 (1.9)	32 (60.4)	7 (13.2)	18 (34.0)
	Thrombocytopenia	7 (13.5)	0	0	23 (43.4)	8 (15.1)	7 (13.2)
	WBC count decreased	7 (13.5)	2 (3.8)	0	6 (11.3)	4 (7.5)	1 (1.9)
	Platelet count decreased	5 (9.6)	1 (1.9)	0	13 (24.5)	2 (3.8)	4 (7.5)
	Leukopenia	4 (7.7)	1 (1.9)	0	14 (26.4)	5 (9.4)	1 (1.9)
	Neutrophil count decreased	3 (5.8)	1 (1.9)	0	11 (20.8)	2 (3.8)	6 (11.3)
Nonhaematological, n (%)^a^	Nausea	20 (38.5)	0	0	18 (34.0)	1 (1.9)	0
	Fatigue	16 (30.8)	1 (1.9)	0	20 (37.7)	2 (3.8)	0
	Dizziness	9 (17.3)	0	0	9 (17.0)	1 (1.9)	0
	Diarrhoea	9 (17.3)	1 (1.9)	0	6 (11.3)	0	0
	Dyspnoea	8 (15.4)	3 (5.8)	0	12 (22.6)	3 (5.7)	0
	Asthenia	8 (15.4)	4 (7.7)	0	9 (17.0)	2 (3.8)	0
	Pyrexia	8 (15.4)	0	0	5 (9.4)	0	0
	Headache	8 (15.4)	0	0	5 (9.4)	0	0
	Alopecia	7 (13.5)	0	0	18 (34.0)	0	0
	Pneumonia	7 (13.5)	3 (5.8)	0	8 (15.1)	7 (13.2)	0
	Cough	7 (13.5)	1 (1.9)	0	8 (15.1)	0	0
	Pruritus	7 (13.5)	0	0	3 (5.7)	0	0
	Vomiting	6 (11.5)	0	0	5 (9.4)	0	0
	Urinary tract infection	6 (11.5)	1 (1.9)	0	3 (5.7)	1 (1.9)	0
	Aspartate aminotransferase increased	6 (11.5)	1 (1.9)	0	2 (3.8)	0	0
	Constipation	5 (9.6)	0	0	12 (22.6)	1 (1.9)	0
	Dehydration	5 (9.6)	0	0	11 (20.8)	3 (5.7)	0
	Noncardiac chest pain	5 (9.6)	1 (1.9)	0	8 (15.1)	1 (1.9)	0
	Decreased appetite	4 (7.7)	1 (1.9)	0	9 (17.0)	0	0
	Hypothyroidism	3 (5.8)	0	0	7 (13.2)	0	0
	Hyperthyroidism	2 (3.8)	0	0	6 (11.3)	0	0

*Notes:* Safety analysis population. Data represent any‐grade AEs occurring in ≥10% of patients in either treatment group during the overall treatment period. Data are listed by frequency in trilaciclib group.

Abbreviations: AE, adverse event; E/P/A, etoposide, carboplatin and atezolizumab; WBC, white blood cell.

^a^ AEs are presented by preferred term.

Serious AEs were reported in 17 (32.7%) patients treated with trilaciclib and 25 (47.2%) patients treated with placebo. One (1.9%) serious AE (Grade 2 deep vein thrombosis) was considered related to trilaciclib. A total of six patients (two in the trilaciclib group and four in the placebo group) had fatal AEs, including failure to thrive, pneumonia (two patients), sepsis, infectious pleural effusion and haemoptysis. Only the AE of infectious pleural effusion was considered by the investigator as related to E/P; none of the fatal AEs were considered related to trilaciclib.

### 3.5 Antitumour efficacy

Among patients evaluable for response, ORR (95% CI) was 56.0% (41.3, 70.0) in the trilaciclib group and 63.5% (49.0, 76.4) in the placebo group. Median (95% CI) DOR was 5.6 (4.4, 7.0) months in the trilaciclib group and 4.3 (3.4, 4.7) months in the placebo group.

**FIGURE 3:**
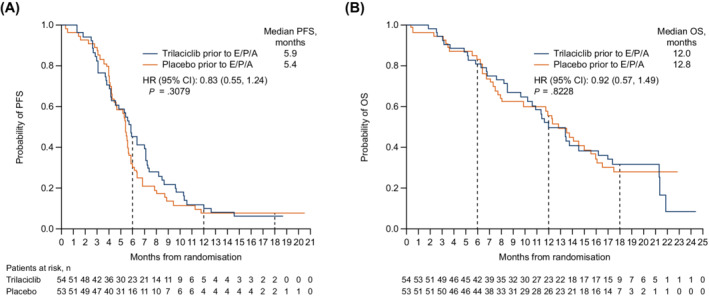
Antitumour efficacy. Kaplan‐Meier estimates of A, Probability of progression‐free survival and B, Probability of overall survival. Data are from the overall treatment period. OS data are not yet mature. E/P/A, etoposide, carboplatin and atezolizumab; HR, hazard ratio; OS, overall survival; PFS, progression‐free survival

Median PFS (95% CI) was 5.9 (4.2, 7.1) months with trilaciclib and 5.4 (4.3, 5.7) months with placebo (HR = 0.83 [95% CI: 0.55, 1.24]) (Figure 3A). As of June 28, 2019, 63% of patients in the trilaciclib group and 66% of patients in the placebo group had died. Median OS (95% CI) was 12.0 (9.6, 16.2) months in patients receiving trilaciclib and 12.8 (7.9, 15.5) months in patients receiving placebo (HR = 0.92 [95% CI: 0.57, 1.49]) (Figure 3B).

### 3.6 Immunomodulatory effects

**FIGURE 4:**
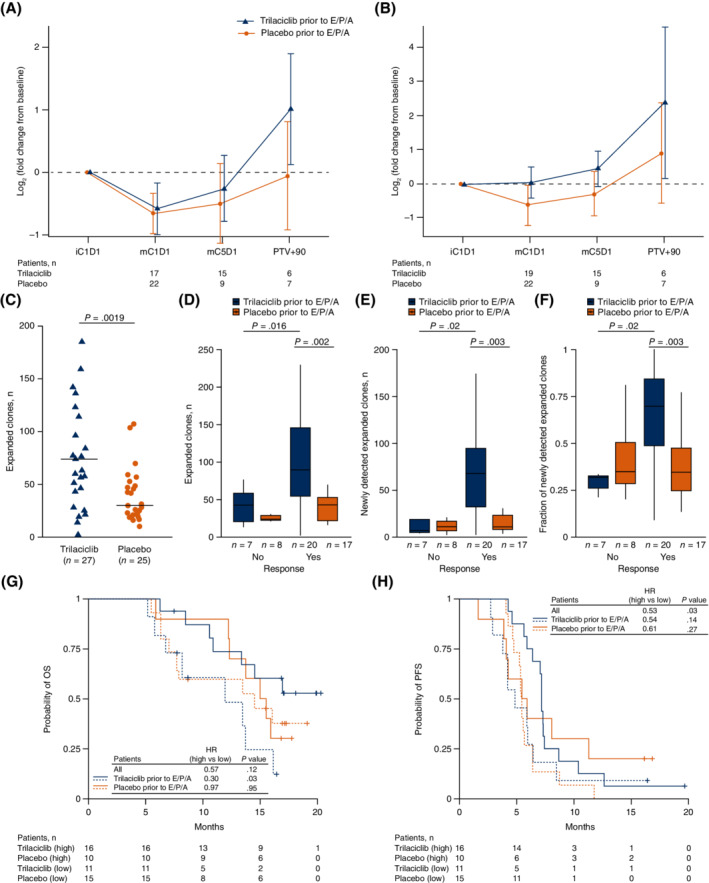
Flow cytometry and T‐cell receptor immunosequencing analysis. A and B, T‐cell populations in whole blood were analysed by flow cytometry at the indicated time points. A, CD8+/regulatory T cells and B, activated CD8+/regulatory T cells. Regulatory T‐cell population was defined as CD45+CD25+CD127lowCD3+CD4+. Error bars represent 95% CI. C‐H, Immunosequencing analysis of T‐cell clones. C, Change from baseline in the number of expanded T‐cell clones was determined by differential abundance analysis of T‐cell receptor β sequences in whole blood from patients at baseline (iC1D1) and the start of maintenance (mC1D1). Horizontal bars indicate median number of expanded clones in each group. D‐F, Peripheral expansion assessed in patients receiving trilaciclib or placebo, according to response to treatment. G and H, Patients were stratified by high (equal or above median, solid lines) and low (below median; dashed lines) fraction of newly detected expanded clones (median fraction 0.429 for all patients) for Kaplan‐Meier estimates of probability of OS and probability of PFS. HR indicates ratio of high relative to low. C, cycle; CI, confidence interval; D, day; E/P/A, etoposide, carboplatin and atezolizumab; HR, hazard ratio; i, induction; m, maintenance; OS, overall survival; PFS, progression‐free survival; PVT, posttreatment visit +90 days

Flow cytometric analysis of T‐cell populations showed that compared with placebo, patients receiving trilaciclib had a higher ratio of CD8+ T cells to Tregs and activated CD8+ T cells to Tregs, through PTV +90, although differences between the treatment groups were not statistically significant (Figure 4A,B). Immunosequencing analyses showed that patients in the trilaciclib group had a significantly higher number of expanded T‐cell clones at the end of induction compared with patients receiving placebo (*P* = .019; Figure 4C). Patients in the trilaciclib group with an antitumour response to E/P/A had significantly more clonal expansion than responders who received placebo (*P* = .002) and more than nonresponders who received trilaciclib (*P* = .016; Figure 4D). Responders receiving trilaciclib also had more newly detected expanded clones and a significant increase in the fraction of newly detected expanded clones (vs total expanded clones) compared with responders in the placebo group (*P* = .003, Figure 4E,F). Stratification of patients below and above the median fraction of newly detected expanded clones to all expanded clones revealed a nonsignificant trend for patients with higher levels of clonal expansion to have longer OS (HR = 0.57, *P* = .117, Figure 4G). A subgroup analysis of patients receiving trilaciclib or placebo revealed that for patients receiving trilaciclib, higher levels of clonal expansion were associated with significantly longer OS (HR = 0.30, *P* = .029, Figure 4G), with similar but nonsignificant trends in PFS (Figure 4H). Similar benefits were observed when patients were stratified above or below median clonal expansion and newly detected expanded clones (Supplementary Figure S3).

## 4 DISCUSSION

The results of this trial show that trilaciclib administered prior to E/P/A therapy in patients with ES‐SCLC reduced CIM, as demonstrated by an improvement in both of the primary myelosuppression endpoints. These findings are consistent with the Phase II trial of trilaciclib administered prior to E/P in patients with newly diagnosed ES‐SCLC and a Phase II trial of trilaciclib prior to topotecan in patients with previously treated ES‐SCLC.^21^, ^30^ Because both the severity and duration of neutropenia correlate with the risk of FN and infections,^31^, ^32^, ^33^ preventing patients from experiencing SN, as measured by these endpoints, provides clinically meaningful benefit to patients. Notably, older patients have ageing HSPCs that put them at increased risk of CIM.^34^ Subgroup analyses showed that trilaciclib improved DSN in C1 and occurrence of SN irrespective of age, but that the magnitude of the effect was larger in patients aged ≥65 years, suggesting that the myelopreservation benefits of trilaciclib also extend to this more vulnerable patient population.

At present, there is no treatment available that prevents the myelosuppressive effects of chemotherapy before they occur.^21^ Current treatments are lineage specific, in the form of ESAs and RBC transfusions for anaemia, G‐CSF administration for neutropenia and platelet transfusions for thrombocytopenia.^35^, ^36^, ^37^, ^38^ Moreover, such measures are only introduced after HSPCs have been damaged by chemotherapy and do not proactively prevent the occurrence of CIM. More specifically, prophylactic treatment with G‐CSF is generally recommended when the risk of FN is considered to be high (>20%) based on the chemotherapy regimen and patient risk factors, and has been shown to reduce the risk of FN and early mortality without significantly affecting antitumour responses. However, each of these interventions is associated with its own set of associated side effects and/or a burden to patients' quality of life.^35^, ^37^, ^38^, ^39^ In our study, reductions in the use of G‐CSFs, RBC transfusions and ESA administrations all favoured trilaciclib over placebo, indicating a decrease in the risk of clinically significant, multilineage CIM.

While myelopreservation outcomes show that the administration of trilaciclib prior to E/P/A therapy results in less chemotherapy‐induced neutropenia and anaemia, PRO assessments also generally favoured trilaciclib over placebo. Overall, more patients had improvements and fewer had deterioration from baseline in HRQoL measures with trilaciclib vs placebo, especially for FWB, PWB, anaemia and fatigue measures. Patients receiving trilaciclib had a longer median time to confirmed deterioration compared with patients receiving placebo in most of the domains and subscales of FACT‐Anemia and FACT‐L, including a 4.6‐month difference in the median time to confirmed deterioration in the fatigue subscale. The rigour of the current study design (randomised, double‐blind, placebo‐controlled) supports the robustness of these findings and is particularly relevant for endpoints such as PROs that are more susceptible to measurement bias.^40^

Clinical concerns over toxicities associated with CIM often lead to dose reductions and/or delays, which are more frequent in elderly patients and can be associated with poorer therapeutic outcomes.^10^, ^41^, ^42^ While most patients completed all four induction cycles, fewer patients in the trilaciclib group had dose delays or reductions compared with the placebo group, suggesting that the administration of trilaciclib prior to chemotherapy helped facilitate the delivery of chemotherapy according to the standard dose and schedule. Previous studies in patients with SCLC have shown that increasing the relative dose intensity of chemotherapy beyond conventional doses rarely yields significant improvements in response rate or survival.^43^ This is in contrast to extensive evidence supporting the importance of dose intensity in patients with early‐stage or advanced breast cancer.^44^, ^45^, ^46^, ^47^ Interestingly, in a Phase II trial of trilaciclib administered prior to gemcitabine/carboplatin vs gemcitabine/carboplatin alone in patients with triple‐negative breast cancer (TNBC), no significant differences were observed in DSN in C1 or the occurrence of SN; however, patients receiving trilaciclib had longer PFS and significantly longer OS vs chemotherapy alone.^48^ In the TNBC study, the overall duration of exposure was increased and patients receiving trilaciclib received higher cumulative doses of chemotherapy, which may have contributed to the observed improvements in OS.^49^

Importantly, administering trilaciclib prior to E/P/A was not associated with a clinically relevant increase in toxicity. Fewer patients receiving trilaciclib experienced high‐grade AEs compared with placebo, which was primarily due to a reduction in high‐grade haematological AEs attributable to cytotoxic chemotherapy. Consistent with these findings, fewer patients receiving trilaciclib were hospitalised due to CIM or sepsis. Compared with placebo, trilaciclib appeared to be associated with an increased frequency of injection‐site reactions and phlebitis/thrombophlebitis; however, these were all low grade. Interestingly, the rate of alopecia among patients receiving trilaciclib was less than half of that observed in the placebo group (13.5% vs 34.0%); in addition to being consistent with trilaciclib's mechanism of action, this finding is likely of importance to patients due to the emotional distress associated with hair loss. Although caution regarding overinterpretation of these results is advised due to the small number of patients and because the study was not specifically designed to evaluate the effect of treatment on alopecia, these findings warrant further investigation.

In theory, trilaciclib has the potential to improve antitumour efficacy by enabling maintenance of chemotherapy dse intensity, while simultaneously facilitating a more favourable, and less damaged, immune system. Preclinical data showed that the addition of trilaciclib prior to chemotherapy plus ICI regimens, including both PD‐L1 and PD‐1 inhibitors, enhanced antitumour response and OS through the modulation of T‐cell proliferation and the tumour immune microenvironment, and increased effector function.^18^, ^20^, ^50^ Flow cytometry and TCR immunosequencing data from this clinical study are consistent with these findings. Compared with placebo, patients receiving trilaciclib had an increased ratio of total and activated CD8+ T cells to Tregs, and increased peripheral T‐cell clonal expansion, suggesting enhanced T‐cell activation. Furthermore, there was a significant enhancement in newly detected expanded clones among patients receiving trilaciclib compared with placebo, which was stronger among patients with an antitumour response to E/P/A, suggesting that trilaciclib may enhance tumour antigen presentation, a phenomenon that has been observed in preclinical studies with other CDK4/6 inhibitors.^51^ These observations are consistent with those from the Phase II study of patients with ES‐SCLC receiving trilaciclib prior to E/P.^21^ In our study, trilaciclib resulted in a higher proportion of activated or effector CD8+ and CD4+ T cells compared with placebo. In addition, high levels of clonal expansion were associated with improved PFS and numerically longer median OS. Taken together, the data suggest that the addition of trilaciclib at least preserves, if not enhances, T‐cell function during treatment with E/P or E/P/A.^18^

There are a number of potential reasons why the findings in the current study did not translate into significant improvements in antitumour efficacy. The effects of trilaciclib on antitumour efficacy are predicted to be primarily driven by the tumour type, chemotherapy type, and host. Specifically, the tumour type must be sufficiently responsive to chemotherapy such that maintenance of chemotherapy dose intensity is beneficial, the chemotherapy should promote immune activation, and the host must be able to mount an effective cytolytic response against the tumour. ES‐SCLC is a highly aggressive tumour that typically recurs and progresses rapidly despite initial response to chemotherapy; among patients treated with chemotherapy and ICIs, median survival remains just 12 months from diagnosis.^13^ Although SCLC has a high tumour mutation burden, it is not considered particularly immunogenic or sensitive to immune modulation.^12^, ^52^, ^53^ PD‐L1 expression has been reported to be low (<50%) in clinical studies, and in our study, was less than 40%. Moreover, SCLC tumours have reduced expression of major histocompatibility complex class I and class II molecules, a known immune escape mechanism, reflecting a less immunogenic environment.^53^, ^54^

Nevertheless, the median OS of 12 months with trilaciclib prior to E/P/A observed in our study is consistent with that seen with E/P/A in the pivotal IMPower133 study,^13^ indicating that, while there was no improvement in the antitumour efficacy of chemotherapy plus atezolizumab, trilaciclib did not antagonise the effects of E/P/A in patients with ES‐SCLC. This is consistent with previous results indicating that the myelopreservation benefits of trilaciclib are not accompanied by detrimental effects on the efficacy of standard‐of‐care chemotherapy regimens.^21^, ^30^

A limitation of our study is the small sample size, which may have reduced the ability to observe statistically significant treatment effects on secondary myelopreservation measures, such as occurrence of FN AEs, infections and antibiotics usage. However, large treatment effects in these endpoints were not expected, given that patients in both arms could receive supportive care interventions for CIM, apart from in C1 where the use of prophylactic G‐CSF and ESAs was prohibited. As a result, the frequency of these secondary events was expected to be low, and the difference between treatment groups to be small. Furthermore, the small sample size meant that it would only be possible to detect large differences in OS. The observed immune effects of trilaciclib are therefore hypothesis generating and require further investigation.

Overall, the results of our study confirm the myelopreservation benefits of trilaciclib administered prior to chemotherapy for SCLC, as demonstrated by the reduction in clinically significant CIM and a reduction in the use of standard‐of‐care interventions. Consistent with these myelopreservation benefits, trilaciclib was associated with improved HRQoL as measured by PRO endpoints that assessed patients' symptoms and functional limitations associated with cancer and CIM, as well as an improved overall safety profile. Although data suggest an enhancement of immune responses with trilaciclib, more research into how this might translate into clinical benefits in antitumour efficacy is needed. Further studies to investigate the clinical use of trilaciclib prior to chemotherapy plus ICI are therefore warranted, particularly in patients with immunogenic tumour types that are sensitive to immune modulation.

## CONFLICT OF INTEREST

Dr Davey Daniel has received research funding from G1 Therapeutics, Inc and Genentech. Dr Jana Jaal has received research funding from AstraZeneca, and has been an adviser to AstraZeneca, Boehringer Ingelheim and MSD. Dr Lowell Hart has received research funding, consultancy and travel expenses from G1 Therapeutics, Inc. Dr Yili Pritchett and Dr Jessica A. Sorrentino are paid employees and shareowners of G1 Therapeutics, Inc. Dr Shannon R. Morris and Joyce M. Antal were employees of G1 Therapeutics, Inc at the time of manuscript preparation and submission. Dr Vladimer Kuchava, Prof. Igor Bondarenko, Dr Oleksandr Ivashchuk, Dr Sreekanth Reddy, Dr Iveta Kudaba and Dr Amiran Matitashvili have no conflicts of interest to declare. Dr Jerome Goldschmidt has participated in speakers bureau and received honoraria from Amgen and Bristol Myers Squibb.

## ETHICS STATEMENT

The study (NCT03041311) was designed and conducted in compliance with the principles of the Declaration of Helsinki and the Good Clinical Practice guidelines of the International Council for Harmonisation. The study protocol and all study‐related materials were approved by the institutional review board or independent ethics committee of each investigational site (Supplementary Methods). Written informed consent was obtained from each patient before the initiation of study procedures.

## Supporting information


**Data S1**: Supplementary Information

## Data Availability

The data that support the findings of this study are available from the corresponding author upon reasonable request.
