# Supplementary material for: Trilaciclib prior to chemotherapy and atezolizumab in patients with newly diagnosed extensive‐stage small cell lung cancer: A multicentre, randomised, double‐blind, placebo‐controlled Phase II trial
Source: Int J Cancer. 2021 Jan 12;148(10):2557–70. doi: 10.1002/ijc.33453 (PMC8048941; doi:10.1002/ijc.33453)
Supplement: Supplementary file 1 — Data S1: Supplementary Information [file IJC-148-2557-s001.pdf]

Title

Trilaciclib prior to chemotherapy and atezolizumab in patients with newly diagnosed extensive-stage small cell lung cancer: a multicentre, randomised, double-blind, placebo-controlled phase II trial

Authors

Davey Daniel, Vladimer Kuchava, Igor Bondarenko, Oleksandr Ivashchuk, Sreekanth Reddy, Jana Jaal, Iveta Kudaba, Lowell Hart, Amiran Matitashvili, Yili Pritchett, Shannon R. Morris, Jessica A. Sorrentino, Joyce M. Antal and Jerome Goldschmidt

Table of Contents

Content	Page
Supplementary Materials & Methods	2–6
Supplementary Tables	7–9
Supplementary Figures	10–12
Supplementary References	13

Supplementary Materials & Methods

Inclusion criteria

- Age ≥18 years
- Unequivocally confirmed diagnosis of small cell lung cancer (SCLC) by histology or cytology, preferably including the presence of neuroendocrine features by immunohistochemistry
- Extensive-stage SCLC
- At least 1 target lesion that was measurable by Response Evaluation Criteria in Solid Tumors, Version 1.1 (RECIST v1.1)
- Haemoglobin ≥9.0 g/dL
- Absolute neutrophil count ≥1.5 × 10^9/L
- Platelet count ≥100 × 10^9/L
- Creatinine ≤1.5 mg/dL or glomerular filtration rate of ≥60 mL/minute
- Total bilirubin ≤1.5 × upper limit of normal (ULN); <3 × ULN if the patient has documented Gilbert's disease
- Aspartate aminotransferase (AST) and alanine transaminase (ALT) ≤2.5 × ULN; ≤5 × ULN in the presence of liver metastases
- Eastern Cooperative Oncology Group performance status (ECOG PS) of 0 to 2
- Predicted life expectancy of ≥3 months
- Contraception:
    - For females:
        - All females of childbearing potential must have had a negative serum beta human chorionic gonadotropin test result at screening. Females must have been either postmenopausal, surgically sterile, or agreed to use 2 forms of highly effective contraception during the study and for 6 months following discontinuation of study treatment.
        - Postmenopausal defined as: (1) at least 60 years of age; (2) medically confirmed ovarian failure; or (3) younger than 60 years of age and have had cessation of regular menses for at least 12 consecutive months with no alternative pathological or physiological cause and/or serum levels of estradiol and follicle-stimulating hormone within the laboratory's reference range for postmenopausal females.
        - Acceptable surgical sterilisation techniques were complete or partial hysterectomy, bilateral tubal ligation with surgery at least 6 months prior to

dosing, or bilateral oophorectomy with surgery at least 2 months prior to dosing.

    - Highly effective methods of contraception were those that resulted in a low failure rate (i.e., less than 1% per year) when used consistently and correctly. These included the following:
        - Established use of oral, injected, or implanted hormonal methods of contraception (stable dose at least 3 months prior to dosing).
        - Placement of an intrauterine device or system.
        - Barrier methods of contraception: condom or occlusive cap (diaphragm or cervical/vault caps) with spermicidal foam/gel/film/cream/suppository. Barrier methods alone (without spermicide) are not acceptable methods. Likewise, spermicide alone is not an acceptable method.
        - Male sterilisation (with the appropriate postvasectomy documentation of the absence of sperm in the ejaculate). For female patients on the study, the vasectomised male partner should be the sole partner for that patient.
        - True abstinence, when this is in line with the preferred and usual lifestyle of the patient. Periodic abstinence (e.g., calendar, ovulation, symptothermal, postovulation methods) and withdrawal are not acceptable methods of contraception.
  - For males:
    - Males must have been surgically sterile or had a female partner who was either postmenopausal, surgically sterile, or using 2 forms of highly effective contraception as noted above. Acceptable surgical sterilisation techniques were vasectomy with surgery at least 6 months prior to dosing. Males must also have refrained from sperm donation during the study and for 6 months following discontinuation of treatment.
- Able to understand and sign an informed consent.

Exclusion:

- Limited-stage SCLC.
- Prior chemotherapy for limited- or extensive-stage SCLC.

- Prior treatment with immunotherapies, including, but not limited to, cluster of differentiation 137 agonists or immune checkpoint blockade therapies (such as anti–PD-1, anti–PD-L1 and cytotoxic T-lymphocyte–associated protein 4 therapeutic antibodies).
- Presence of symptomatic brain metastases requiring immediate treatment with radiation therapy or steroids.
- Malignancies other than SCLC within 3 years prior to randomisation, with the exception of those with a negligible risk of metastasis or death who have been treated with expected curative outcome.
- History of idiopathic pulmonary fibrosis, organising pneumonia, drug-induced pneumonitis, idiopathic pneumonitis, or evidence of active pneumonitis on screening chest computed tomography scan (history of radiation pneumonitis in the radiation field [fibrosis] was permitted).
- Active, known, or suspected autoimmune disease that has required systemic treatment in the past 2 years (i.e., with use of disease modifying agents, corticosteroids, or immunosuppressive drugs). Exceptions included vitiligo, controlled asthma, type I diabetes, Graves’ disease, Hashimoto’s disease, or with medical monitor approval. Stable replacement therapy (e.g., thyroxine, insulin, or physiologic corticosteroid replacement therapy for adrenal or pituitary insufficiency) for well-controlled disease was not considered a form of systemic treatment.
- Uncontrolled ischaemic heart disease or uncontrolled symptomatic congestive heart failure (Class III or IV as defined by the New York Heart Association functional classification system).
- Known history of stroke or cerebrovascular accident within 6 months prior to enrollment.
- Serious active infection at the time of enrollment.
- Psychiatric illness/social situations that would limit study compliance.
- Other uncontrolled serious chronic disease or conditions that in the investigator’s opinion could affect compliance or follow-up in the protocol.
- Known human immunodeficiency virus, known active hepatitis B (e.g., hepatitis B surface antigen reactive or hepatitis B virus DNA detected) or hepatitis C (e.g., hepatitis C virus RNA [qualitative] detected).
- Radiotherapy to any site within 2 weeks prior to enrollment.
- Receipt of any investigational medication within 4 weeks prior to enrollment.
- Administration of a live attenuated vaccine within 4 weeks before enrollment or anticipation that such a live attenuated vaccine will be required during the study.

- Influenza vaccination should be given during influenza season only (approximately October to March). Patients must not receive live, attenuated influenza vaccine (e.g., FluMist) within 4 weeks prior to enrollment, at any time during the study and at least 5 months after the last dose of atezolizumab.
- Patients with a condition requiring systemic treatment with either corticosteroids (>10 mg daily prednisone equivalents) or other immunosuppressive medications (including but not limited to cyclophosphamide, azathioprine, methotrexate, thalidomide and antitumor necrosis factor agents) within 14 days of study drug administration. Inhaled or topical steroids and adrenal replacement doses >10 mg daily prednisone equivalents are permitted in the absence of active autoimmune disease.
- Hypersensitivity to any of the components of the formulation of etoposide or etoposide phosphate.
- Hypersensitivity to carboplatin or other platinum-containing compounds or to mannitol.
- History of severe allergic, anaphylactic, or other hypersensitivity reactions to chimeric or humanised antibodies or fusion proteins.
- Known hypersensitivity to Chinese hamster ovary cell products or other recombinant human antibodies.
- Legal incapacity or limited legal capacity.
- Pregnant or lactating women.

Flow cytometry analysis

The estimated proportion of activated CD8+ T cells (CD38+HLADR+CD3+CD8+), activated Th1 cells (CXCR3+CXCR6-CD38+HLADR+CD3+CD4+) and regulatory T cells (CD45+CD25+CD127lowCD3+CD4+) in peripheral blood mononuclear cells (PBMC) was calculated as previously described.[1, 2] To assess the effect of trilaciclib on lymphocyte levels over time, a linear mixed-effect model was fitted to longitudinal data. The analysis used an unstructured variance–covariance structure, according to the following model: Test Result ~ Weeks + Treatment + Weeks : Treatment + PatientID (random effect).

T-cell receptor analysis

T-cell receptor β CDR3 regions were amplified and sequenced from purified genomic DNA in human PBMC using the immunoSEQ® Assay (Adaptive Biotechnologies, Seattle, WA, USA). Expanded T-cell clones (defined as frequency increased in posttreatment versus pretreatment samples in a given patient) were computationally identified as previously described.[3] *p* values were computed using a Wilcoxon Rank Sum test. Survival was assessed using survival and survminer R packages, with Cox

proportional hazard regression analysis. A binomial model with Benjamini-Hochberg correction for multiple comparisons at the amino acid level was used for the identification of clones with significantly different frequencies.

Independent Ethics Committees and Review Boards

Advarra IRB (Central IRB), Columbia, MD
Comite Autonomico de Etica de la Investigacion de Galicia, A Coruna, Spain
CPP llc-de France VII, Hopital de Bicetre, Portail-des champs-Sectur Bleu, Le Kremlin Bicetre, France
Ethics Committee for Clinical Trials, Riga, Latvia
Ethics Committee for Multicenter Trials, Sofia, Bulgaria
Ethics Committee of Chernivtsi Regional Clinical Oncology Center, Chernivtsi, Ukraine
Ethics Committee of Dnipropetrovsk City Multispecialty Clinical Hospital no. 4, Dnipro, Ukraine
Ethics Committee of Sumy Regional Clinical Oncology Center, Sumy, Ukraine
Ethics Committee of Ternopil Regional Clinical Oncology Center, Ternopil, Ukraine
Ethics Committee of Volyn Regional Oncology Center, Lutsk, Ukraine
Independent Ethics Committee within LTD Cancer Research Centre, Tiblisi, Georgia
Independent Ethics Committee within LTD Institute of Clinical Oncology, Tiblisi, Georgia
Independent Ethics Committee within LTD Institute for Personalized Medicine, Tiblisi, Georgia
Independent Ethics Committee within LTD Research Institute of Clinical Medicine, Tiblisi, Georgia
Tallinn Medical Research Ethics Committee, Tallinn, Estonia
University of Miami Institutional Review Board, Miami, FL
Western Institutional Review Board, Puyallup, WA

Supplementary Tables

Supplementary Table S1. Myelopreservation efficacy results.

Endpoints		Trilaciclib prior to E/P/A (*n* = 54)	Placebo prior to E/P/A (*n* = 53)	Treatment effect[a] (mean difference or relative risk) (95% CI)	Adjusted one-sided *p* value[b]
**Primary endpoints**	Mean DSN in Cycle 1, days (SD)	0 (1.0)	4 (4.7)	–3.6[c] (–4.9, –2.3)	<0.0001
	Occurrence of SN, n (%)	1 (1.9)	26 (49.1)	0.038 (0.008, 0.195)	<0.0001
**Key secondary endpoints**	All-cause dose reductions, event rate per cycle, n	0.021	0.085	0.242 (0.079, 0.742)	0.0195
	Patients with RBC transfusion on/after week 5, n (%)	7 (13.0)	11 (20.8)	0.642 (0.294, 1.404)	0.1335
	Patients with G-CSF administration, n (%)	16 (29.6)	25 (47.2)	0.646 (0.403, 1.034)	0.0686
				**Adjusted rate ratio (95% CI)**	***p* value**
**Supportive secondary endpoints**	Patients with FN AEs, n (%)	1 (1.9)	3 (5.7)	–	NE
	Cycles with G-CSF administration/total number of cycles (incidence rate per cycle)	29/195 (0.15)	56/200 (0.28)	–	–
	Patients with ESA administration, n (%)	3 (5.6)	6 (11.3)	0.517 (0.137, 1.957)	0.3316
	Patients with grade 3/4 decreased haemoglobin, n (%)	10 (18.5)	15 (28.3)	0.709 (0.358, 1.405)	0.3243
	Patients with platelet transfusion, n (%)	1 (1.9)	2 (3.8)	–	NE
	Patients with grade 3/4 decreased platelet counts, n (%)	1 (1.9)	20 (37.7)	0.053 (0.008, 0.356)	0.0026
	Patients with IV antibiotic administration, n (%)	10 (18.5)	12 (22.6)	0.916 (0.430, 1.950)	0.8196
	Patients with infection SAEs, n (%)	3 (5.6)	7 (13.2)	0.477 (0.130, 1.752)	0.2644
	Patients with pulmonary infection SAEs, n (%)	2 (3.7)	5 (9.4)	–	NE
	Patients with chemotherapy dose reductions, n (%)				
	Etoposide	3 (5.8)	14 (26.4)		
	Carboplatin	1 (1.9)	13 (24.5)	–	–

Abbreviations: AEs, adverse events; ANCOVA, analysis of covariance; DSN, duration of severe neutropenia; ECOG PS, Eastern Cooperative Oncology Group performance status; ESA, erythropoiesis-stimulating agent; FN, febrile neutropenia; G-CSF, granulocyte colony-stimulating factor; IV, intravenous; NE, not evaluable; RBC, red blood cell; SAE, serious adverse event; SD, standard deviation; SN, severe neutropenia.

[a]The following statistical models were used to assess treatment effects: nonparametric ANCOVA (DSN in Cycle 1); modified Poisson regression (occurrence of SN and RBC transfusion on/after 5 week); negative binomial regression (number of all-cause dose reduction). All models included the following as covariates: ECOG PS, presence of brain metastases and the corresponding baseline laboratory values.

[b]One-sided adjusted *p* value obtained from a Hochberg-based gatekeeping procedure.

[c]Mean difference.

Supplementary Table S2. Subgroup analyses by age.

Endpoint	< 65 years of age			≥65 years of age		
	**Trilaciclib (n = 27)**	**Placebo (n = 27)**	***p* value**	**Trilaciclib (n = 27)**	**Placebo (n = 26)**	***p* value**
DSN in C1, mean (SD)	0 (1.3)	2 (3.9)	0.0068	0 (0.0)	5 (5.1)	<0.0001
Occurrence of SN, n (%)	1 (3.7)	9 (33.3)	0.0052	0 (0.0)	17 (65.4)	<0.0001
Patients with RBC transfusions on or after week 5, n (%)	2 (7.4)	1 (3.7)	NE	5 (18.5)	10 (38.5)	0.1707

Abbreviations: C, cycle; DSN, duration of severe neutropenia; NE, not evaluable; RBC, red blood cell; SD, standard deviation; SN, severe neutropenia.

Supplementary Table S3. Number of hospitalisations (any cause or due to chemotherapy-induced myelosuppression or sepsis)

Endpoints	Trilaciclib prior to E/P/A (*n* = 52)	Placebo prior to E/P/A (*n* = 53)	*p* value
Patients hospitalised due to any cause, n (%)	12 (23.1)	14 (26.4)	–
Incidence of hospitalisation due to any cause, event rate (per 100 cycles)	10.77	12.50	–
Patients hospitalised due to CIM or sepsis, n (%)	2 (3.8)	6 (11.3)	0.1287
Incidence of hospitalisation due to CIM or sepsis, event rate (per 100 cycles)	1.03	5.50	–
Patients hospitalised due to CIM, n (%)	2 (3.8)	5 (9.4)	0.2205
Incidence of hospitalisation due to CIM, event rate (per 100 cycles)	1.03	4.50	–
Patients hospitalised due to sepsis, n (%)	0	2 (3.8)	–
Incidence of hospitalisation due to sepsis, event rate (per 100 cycles)	–	1.00	–

Abbreviations: E/P/A, etoposide, carboplatin, and atezolizumab; CIM, chemotherapy-induced myelosuppression.

Supplementary Figures

Supplementary Figure S1. Patient disposition

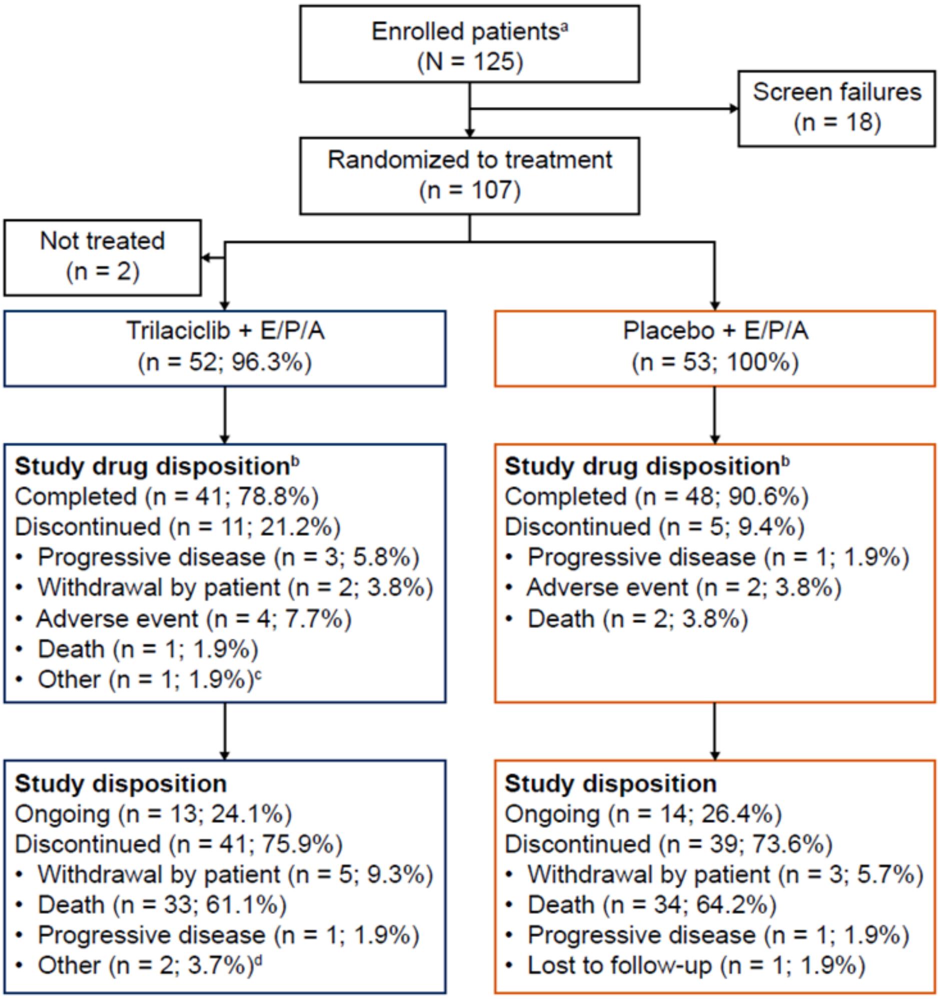

Abbreviations: E/P/A, etoposide, carboplatin, and atezolizumab.

[a]Enrolled patients were those who signed informed consent.

[b]During induction, defined as completing 4 cycles.

[c]One patient discontinued study drug due to reason of "Other: intercurrent illness/prolonged hospitalisation".

[d]Two patients were randomised in error without receiving study drugs; therefore, their reason for discontinuing the study is indicated as "Other".

Supplementary Figure S2. Haematology assessments

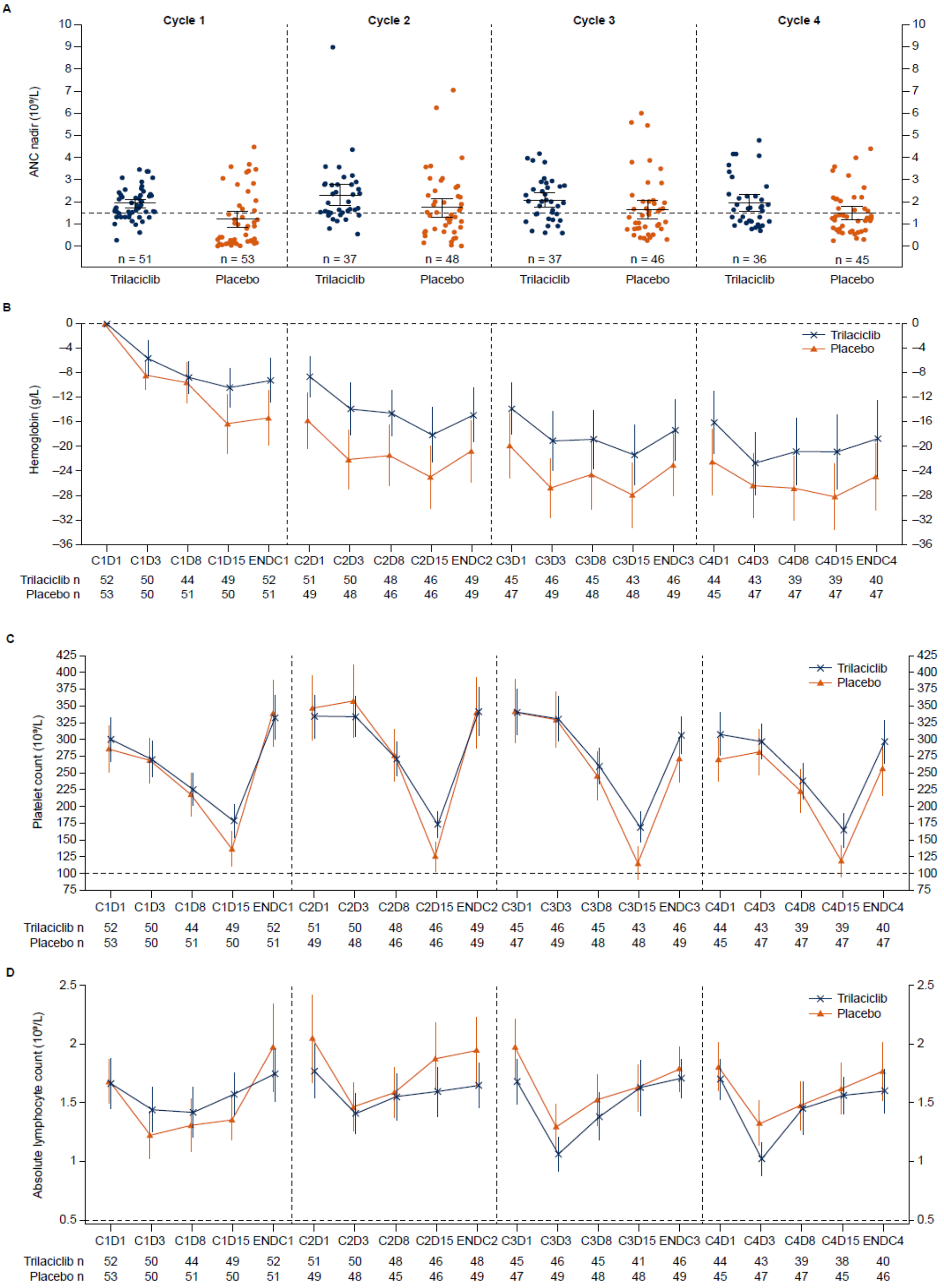

Abbreviations: ANC, absolute neutrophil count; C, cycle; D, day; ENDC, end of cycle (defined as the last value measured prior to the first day of dosing in the subsequent cycle); CI, confidence interval; L, litre; g, gram.

(**A**) Mean ANC nadir values by cycle. Dots represent nadir per patient. (**B**) Mean change from baseline in haemoglobin by windowed visit. (**C**) Mean platelet count by windowed visit. (**D**) Mean absolute lymphocyte count by windowed visit. Data are from the induction phase and show mean counts with 95% CI.

Supplementary Figure S3. Immunosequencing data

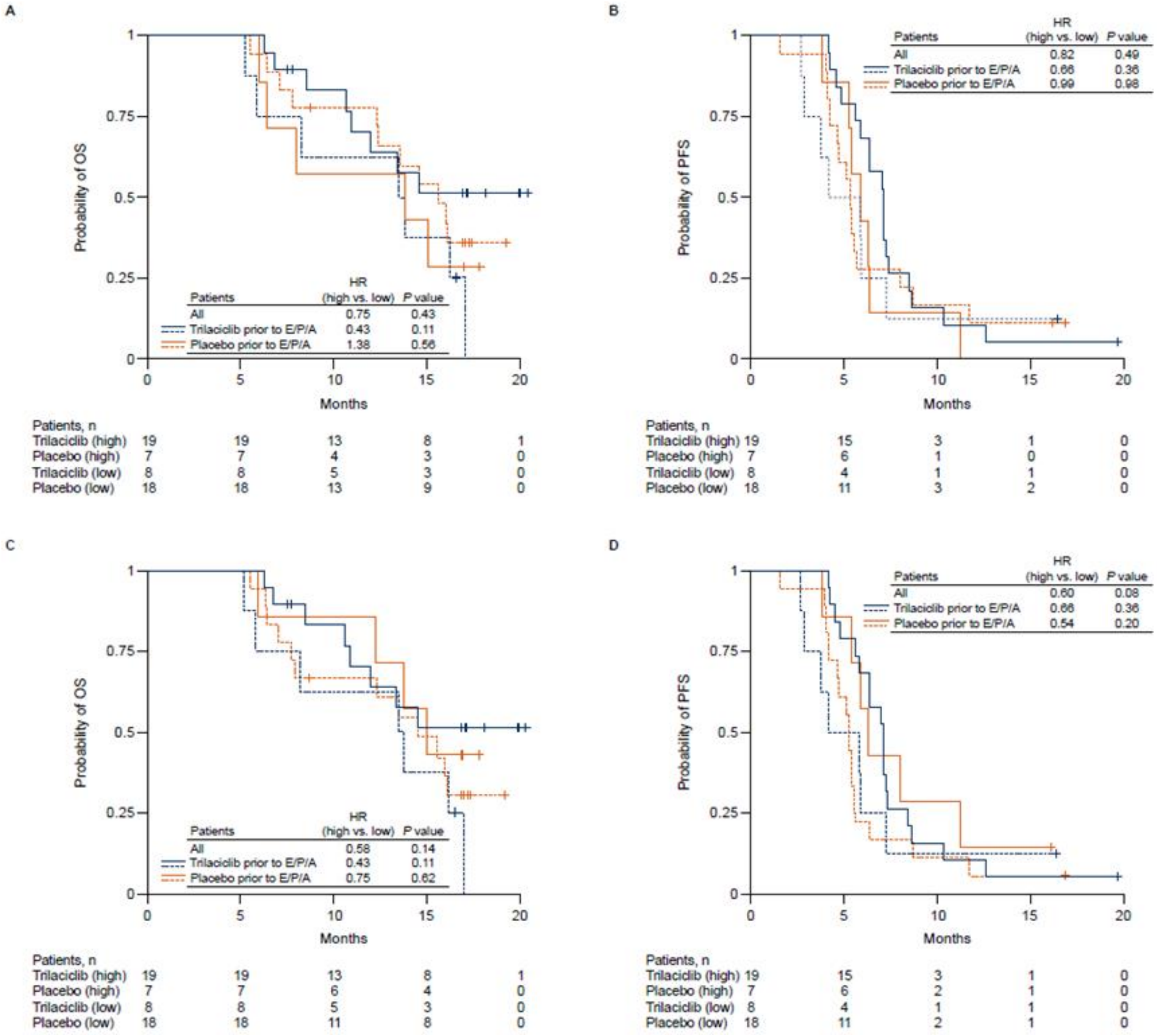

Abbreviations: E/P/A, etoposide, carboplatin and atezolizumab; HR, hazard ratio; OS, overall survival; PFS, progression-free survival.

For Kaplan–Meier estimates of probability of overall survival and probability of PFS, (**A, B**) patients were stratified by high (equal or above median, solid lines) and low (below median; dashed lines) number of expanded T-cell clones (median 48 clones for all patients) and (**C, D**) high (equal or above median, solid lines) and low (below median; dashed lines) number of newly detected expanded T-cell clones (median 19 clones for all patients). HR indicates ratio of high relative to low.

Supplementary References

1. Tumeh PC, Harview CL, Yearley JH, Shintaku IP, Taylor EJ, Robert L, Chmielowski B, Spasic M, Henry G, Ciobanu V, West AN, Carmona M, et al. PD-1 blockade induces responses by inhibiting adaptive immune resistance. *Nature* 2014;515:568-71.
2. Hsu M, Sedighim S, Wang T, Antonios JP, Everson RG, Tucker AM, Du L, Emerson R, Yusko E, Sanders C, Robins HS, Yong WH, et al. TCR sequencing can identify and track glioma-infiltrating T cells after DC vaccination. *Cancer Immunol Res* 2016;4:412-8.
3. DeWitt WS, Emerson RO, Lindau P, Vignali M, Snyder TM, Desmarais C, Sanders C, Utsugi H, Warren EH, McElrath J, Makar KW, Wald A, et al. Dynamics of the cytotoxic T cell response to a model of acute viral infection. *J Virol* 2015;89:4517-26.
